# 

**Published:** 1897-05

**Authors:** 


					﻿ATLANTA
IWEDIGflli AND SDRGIGflh
________JOURNAL.____________
new sebies. Atlanta, Ga., May, 1897.	No. 3.

				

